# Tandem sialoglycan-binding modules in a *Streptococcus sanguinis* serine-rich repeat adhesin create target dependent avidity effects

**DOI:** 10.1074/jbc.RA120.014177

**Published:** 2020-08-20

**Authors:** Haley E. Stubbs, Barbara A. Bensing, Izumi Yamakawa, Pankaj Sharma, Hai Yu, Xi Chen, Paul M. Sullam, T. M. Iverson

**Affiliations:** 1 Graduate Program in Chemical and Physical Biology, Vanderbilt University, Nashville, Tennessee, USA; 2 Department of Medicine, Veterans Affairs Medical Center, San Francisco, California, USA; 3 Department of Medicine, University of California, San Francisco, California, USA; 4 Department of Pharmacology, Vanderbilt University, Nashville, Tennessee, USA; 5 Department of Chemistry, University of California, Davis, California, USA; 6 Department of Biochemistry, Vanderbilt University, Nashville, Tennessee, USA; 7 Center for Structural Biology, Vanderbilt University, Nashville, Tennessee, USA

**Keywords:** adhesin, bacterial adhesion, bacterial pathogenesis, carbohydrate-binding protein, crystal structure, infectious disease, protein crystallization, Streptococcus, X-ray crystallography, host-pathogen interaction

## Abstract

Sialic acid–binding immunoglobulin-like lectins (Siglec)–like domains of streptococcal serine-rich repeat (SRR) adhesins recognize sialylated glycans on human salivary, platelet, and plasma glycoproteins via a YTRY sequence motif. The SRR adhesin from *Streptococcus sanguinis* strain SK1 has tandem sialoglycan-binding domains and has previously been shown to bind sialoglycans with high affinity. However, both domains contain substitutions within the canonical YTRY motif, making it unclear how they interact with host receptors. To identify how the *S. sanguinis* strain SK1 SRR adhesin affects interactions with sialylated glycans and glycoproteins, we determined high-resolution crystal structures of the binding domains alone and with purified trisaccharides. These structural studies determined that the ligands still bind at the noncanonical binding motif, but with fewer hydrogen-bonding interactions to the protein than is observed in structures of other Siglec-like adhesins. Complementary biochemical studies identified that each of the two binding domains has a different selectivity profile. Interestingly, the binding of SK1 to platelets and plasma glycoproteins identified that the interaction to some host targets is dominated by the contribution of one binding domain, whereas the binding to other host receptors is mediated by both binding domains. These results provide insight into outstanding questions concerning the roles of tandem domains in targeting host receptors and suggest mechanisms for how pathogens can adapt to the availability of a range of related but nonidentical host receptors. They further suggest that the definition of the YTRY motif should be changed to ϕTR*X*, a more rigorous description of this sialic acid–recognition motif given recent findings.

The serine-rich repeat (SRR) adhesins are a family of bacterial cell-surface glycoproteins containing two sequence motifs where serine constitutes ∼50% of the sequence (Fig. 1 and Fig. S1). These adhesins follow a modular architecture that initiates with an atypical signal peptide, a short N-terminal serine-rich region, a ligand-binding region (frequently termed “adhesin_BR_” or “strain_BR_,” *e.g.* the binding region from *Streptococcus sanguinis* strain SK1 is termed SK1_BR_), a second serine-rich repeat region that varies in length between several hundred and several thousand amino acids with serine as every other residue, *e.g.* …SVSASTSASTSASTSAS…, and a cell wall anchoring motif. Fiber diffraction studies suggest that these repeat regions form a spring-like linker that tethers the host binding region to the bacterium (1).

**Figure 1.**
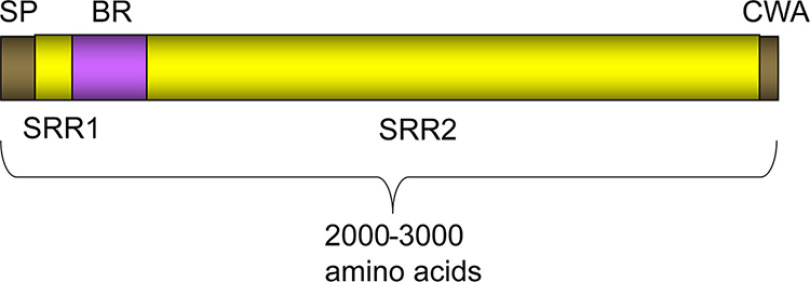
**General organization of SRR adhesin proteins.** SRR adhesins initiate with a ∼90-amino acid N-terminal signal peptide (*SP*) that facilitates trafficking to a specialized glycoprotein transporter known as the accessory Sec system. The serine-rich repeat regions (*SRR1* and *SRR2*) are extensively *O*-glycosylated in the bacterial cytoplasm prior to transport. The ligand-binding region (*BR*) varies depending upon the organism and contains structural modules that are highly diverse in sequence, fold, and function, which may provide binding specificity for different bacterial strains (6). The C-terminal cell wall anchor (*CWA*) includes an LP*X*TG sequence motif that covalently links the cell wall peptidoglycan.

The SRR adhesins are expressed by a variety of Gram-positive commensal and pathogenic bacteria and are broadly distributed (2). A survey of NCBI GenBank^TM^ identified over a thousand sequences that may belong to this family. Indeed, all sequenced strains of *Streptococcus gordonii* and *S. sanguinis* encode serine-rich repeat adhesins (3), and homologs have been found in strains of *Streptococcus oralis* and *Streptococcus mitis*, as well as other oral streptococci (2, 4–6). One known functional role of these SRR adhesins is to mediate attachment to protein or glycoprotein receptors, which allows for adherence to host tissues. Accordingly, SRR adhesins have been linked to a variety of infections, including endocarditis, meningitis, and pneumonia (7–12).

Despite the conserved functional organization (Fig. 1), the ligand-binding regions are highly diverse with species-specific trends in the binding region type. Some of the ligand-binding regions of SRR adhesins target glycan structures (13). For example, the SRR adhesins of *S. gordonii* and *S. sanguinis* bind *O*-linked sialoglycans displayed on mucin-like proteins including salivary glycoprotein MUC7 and platelet glycoprotein GPIb (2, 14, 15). Binding to MUC7 may facilitate oral colonization, whereas interaction with platelet GPIb can allow streptococci to establish endocardial infections (14, 16).

Interaction of the *S. gordonii* and *S. sanguinis* SRR adhesins with host sialoglycan structures relies upon a domain within the binding region related in fold to mammalian sialic acid–binding immunoglobulin-like lectins (Siglecs); indeed both are organized around a V-set Ig fold (6, 16–18). These “Siglec-like” SRR adhesins always contain a second domain immediately following the Siglec domain (16). Termed the Unique domain, this C-terminal region has no counterpart in mammalian Siglecs, and its function remains unknown.

Despite the conserved fold in the Siglec domain, the binding location for sialoglycans differs between the bacterial Siglec-like adhesins and mammalian Siglecs. The streptococcal Siglec-like adhesins hydrogen-bond with sialic acid via a semiconserved YTRY sequence motif on the F strand of the V-set Ig fold (Fig. 2) (6, 16, 17, 19). Of these, the first Tyr residue contributes only backbone interactions to the ligand. Here, the aromatic side chain faces away from the binding site and is involved in packing interactions that likely contribute to the correct presentation of central Thr-Arg. Thr-Arg makes multiple key side-chain hydrogen-bonding contacts to the sialic acid of host sialoglycans, and therefore the sequence of these central residues appears to be the most important for binding (16, 17). Prior mutagenesis of either the Thr or Arg in characterized Siglec-like adhesins substantially reduces binding to defined, synthetic sialoglycans and to platelets (6, 19, 20). Moreover, isogenic strains of streptococci containing mutations in the YTRY motif exhibit reduced virulence in an animal model (21). The final Tyr of the motif contributes a single hydrogen bond to the subterminal galactose of α2,3-sialoglycans and is therefore not involved in sialic acid recognition but may contribute to overall binding affinity of sialoglycans (16).

**Figure 2.**
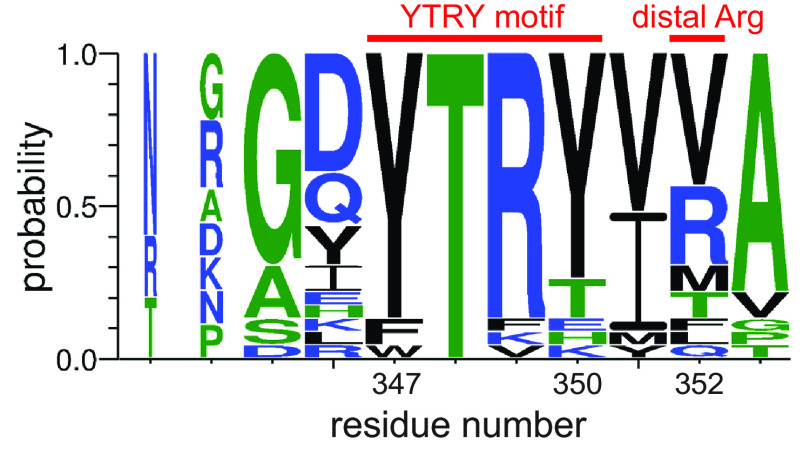
**The sialoglycan-binding motif of Siglec-like SRR adhesins.** The conservation of residues is indicated by *letter size* with the *larger letters* representing a more strongly conserved residue. The positions of the YTRY motif (positions 347–350) and the distal Arg (position 352) are notated with a *red line* above the *letters*. The numbering reflects the residue positions within SK1_Siglec1_. The *letters* colored *blue*, *green*, and *black* indicate charged, nonpolar, and polar residues, respectively. The adhesins included in the alignment are WP_125444382.1 from *S. gordonii* strain M99, WP_046165954.1 from *S. gordonii* strain 72-40, WP_080889728.1 from *S. gordonii* strain G9B, WP_046165954.1 from *Streptococcus sp.* strain 1236FAA, WP_009659981.1 from *Streptococcus sp.* strain AS14, WP_002906900.1 from *S. sanguinis* strain SK115, WP_125439128.1 from *S. sanguinis* strain SK150, WP_125444035.1 from *S. sanguinis* strain SK678, WP_081102781.1 from *S. gordonii* strain Challis, WP_045635027.1 from *S. gordonii* strain UB10712, WP_080555651.1 from *S. sanguinis* strain SK1, WP_011836739.1 from *S. sanguinis* strain SK36, WP_080555852.1 from *S. sanguinis* strain SK408, WP_000466180.1 from *S. sanguinis* strain SK140, WP_046165954.1 from *S. sanguinis* strain PS478, WP_087941957.1 from *S. sanguinis* strain SK1056, WP_080557024.1 from *S. sanguinis* strain SK330, WP_080560819.1 from *S. sanguinis* strain SK355, WP_080555460.1 from *S. sanguinis* strain SK405, WP_061600538.1 from *S. gordonii* strain SK49, and WP_000466181.1 from *S. oralis* strain SF100. Although previously termed the YTRY motif, this binding sequence motif is formally defined as ϕTR*X*, where ϕ represents Trp, Phe, or Thr, and *X* represents Tyr, Thr, Glu, His, or Lys.

Recent structural and engineering studies revealed that sialoglycan binding and selectivity are also affected by three adjacent loops of high sequence diversity (18). Using nomenclature from the V-set Ig fold identified them as the CD loop, the EF loop, and the FG loop (16). Amino acid side chains in these loops directly hydrogen-bond with sialoglycan ligands (16, 17, 19), and the sequence diversity of these loops is proposed as a major determinant of sialoglycan selectivity in the Siglec-like adhesins. As a result, they have been termed “selectivity loops” (18).

The only structurally characterized Siglec-like binding region that differs somewhat in the topology of its binding pocket is found in SrpA from *S. sanguinis* strain SK36. In SrpA_BR_, the YTRY sequence is a noncanonical FTRT but retains the central Thr-Arg important for ligand binding (17, 19). In addition, SrpA_BR_ lacks an appreciable FG selectivity loop. Notably, SrpA_BR_ contains a second Arg residue outside of the canonical binding sequence motif (Fig. 2) that cooperates with the noncanonical FTRT sequence to promote sialoglycan binding (17, 19). This residue is not highly conserved in the Siglec-like binding regions (Fig. 2) and is located too far from the FTRT motif to interact with a bound trisaccharide. However, structures of SrpA_BR_ show that the binding pocket is contiguous with this distal arginine both because of the presence of a Thr *versus* Tyr in the fourth position of the YTRY motif and because of the absence of the FG loop. These alterations extend the glycan-binding site, which may allow the accommodation of either two oriented trisaccharides or larger, branched sialoglycans. Indeed, a disialylated hexasaccharide has been modeled into this site with the distal arginine binding to the second sialic acid of this significantly larger and disialylated ligand (17).

The binding region from *S. sanguinis* strain SK1 (residues 252–660 and termed SK1_BR_) differs from structurally characterized Siglec-containing binding regions in two ways (6). First, it contains two copies of the Siglec and Unique domains in tandem (SK1_Siglec1_-SK1_Unique1_-SK1_Siglec2_-SK1_Unique2_) (Fig. S1) with sequence identity/similarity of 39%/56% between the two Siglec domains and 44%/50% between the two Unique domains. Tandem domains are rarely observed in sequences of the Siglec-like adhesins. Only eight other tandem domain Siglec-like binding regions are identifiable in GenBank^TM^. Seven of the eight SRR adhesins have ≥94% identity and ≥96% similarity to SK1_BR_; these adhesins are from various strains of *S. sanguinis* and one from *Streptococcus cristatus* (Fig. S1). The other adhesin containing a tandem domain is FapC from *S. oralis* subsp. *dentisani* strain F0392 (Fig. S1) (4), in which the FapC_BR_-binding region exhibits 25% identity and 39% similarity to SK1_BR_. This evolutionary relatedness is consistent with evidence that FapC_BR_ may bind sialoglycans and may be important for oral colonization (4). The functional implications for tandem domains have not been explained in the literature.

The second way that SK1_BR_ differs from other structurally characterized Siglec-like binding regions is that each of the putative Siglec domains of SK1_BR_ contains a noncanonical YTRY sialic acid–binding sequence motif that lacks the central Thr-Arg deemed to be critical for binding in other Siglec-like adhesins (6). This motif in SK1_Siglec1_ is YTKY, and the motif in SK1_Siglec2_ is YTFK (6). Although it has been shown that SK1_BR_ binds sialoglycans (6), it was unknown whether both Siglec domains could bind sialoglycans. If so, what is the relative contribution of each to binding? How do the noncanonical YTRY sequence motifs, particularly in SK1_Siglec2_, maintain the interaction with host receptors?

Here, we present crystal structures of unliganded and sialoglycan-bound SK1_BR_, which show that both SK1_Siglec1_ and SK1_Siglec2_ adopt V-set Ig folds and that both domains interact with sialoglycan ligands at the noncanonical YTRY motifs. We validate these interactions using binding studies of isolated SK1_Siglec1+Unique1_ and SK1_Siglec2+Unique2_ and demonstrate that each domain has a distinct selectivity profile for synthetic sialoglycans and glycoprotein ligands. The tandem domains allow increased binding to a host salivary glycoprotein, MUC7, via an avidity effect, possibly by binding simultaneously to two oriented, large glycans. In contrast, platelet binding mainly occurs via SK1_Siglec2_, and binding to the plasma glycoprotein PRG4 (also called lubricin) mainly occurs via SK1_Siglec1_. Taken together, these findings support a mechanism of host interaction in which the tandem domains of the *S. sanguinis* strain SK1 adhesin interact most strongly with a patch of oriented large glycans and indicate that each individual domain differently impacts binding to sialoglycoprotein targets. This adhesin architecture therefore allows for increased flexibility and breadth in the host receptors that are recognized.

## Results

### Structure of S. sanguinis SK1_BR_

**Figure 3.**
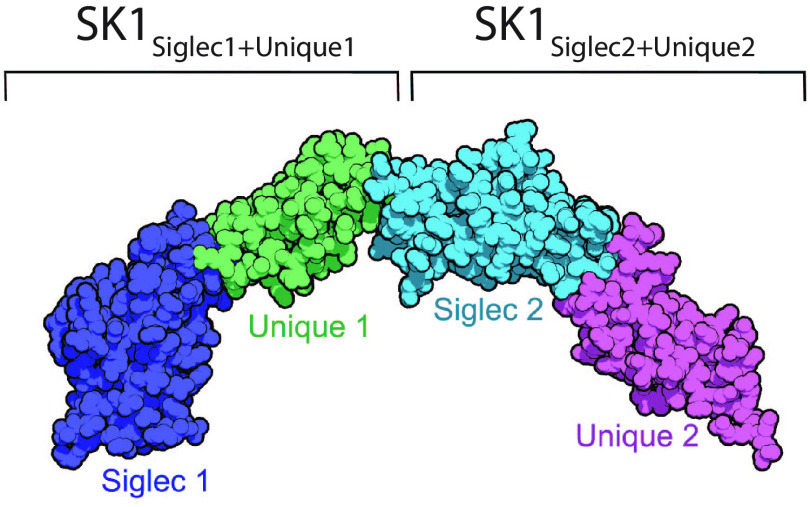
**Structure of the binding region of unliganded SK1.** SK1_BR_ has four domains in the order SK1_Siglec1_, SK1_Unique1_, SK1_Siglec2_, and SK1_Unique2_ depicted from *left* to *right* and colored by domain. The domain repeats, named SK1_Siglec1+Unique1_ and SK1_Siglec2+Unique2_, are homologous but not identical.

To develop hypotheses for how SK1_BR_ binds to sialoglycans, we began by determining its X-ray crystal structure using molecular replacement methods (Tables 1 and 2). The tandem repeats of unliganded SK1_BR_ fold independently, and slight angles between the domains yield an elongated and overall arc shape (Fig. 3). The limits of the domains can be clearly distinguished as SK1_Siglec1_ (residues 252–377), SK1_Unique1_ (residues 378–453), SK1_Siglec2_ (residues 454–573), and SK1_Unique2_ (residues 574–660).

**Table 1:** **Diffraction data collection statistics for the liganded and unliganded SRR-binding region from *S. sanguinis* strain SK1** The values in parentheses are statistics for the highest resolution shell.

	None	sTa	3´sLn
**SBGrid Entry**	756	754	755
Resolution (Å)	2.00	1.55	2.10
Highest resolution shell (Å)	2.00–2.07	1.55–1.58	2.10–2.18
**Data collection**			
Beamline	APS 21-ID-F	APS 21-ID-F	APS 21-ID-F
Wavelength (Å)	0.97872	0.97946	0.97946
Space group	P2_1_2_1_2	P2_1_2_1_2	P2_1_2_1_2
Unit cell dimensions (Å)			
*a*	82.213	83.498	81.549
*b*	269.859	271.85	271.063
*c*	47.511	47.815	46.849
*R*_sym_	0.13 (0.85)	0.067 (0.615)	0.086 (0.514)
*R*_pim_	0.045 (0.285)	0.035 (0.402)	0.045 (0.311)
*I*/σ	25.78 (3.38)	26.6 (1.9)	16.26 (1.51)
Completeness (%)	100 (99.8)	93.1 (74.4)	92.34 (67.30)
Redundancy	10.0 (9.9)	4.3 (2.8)	4.3 (3.3)
CC_1/2_	0.910 (0.706)	0.997 (0.723)	0.999 (0.727)

**Table 2:** **Refinement statistics for the liganded and unliganded SRR-binding region from *S. sanguinis* strain SK1** The Ramachandran statistics were obtained using the MolProbity output of Phenix (40). ASU, asymmetric unit.

	None	sTa	3sLn
**PDB entry**	6VS7	6VT2	6VU6
**Model content (per ASU)**			
Protein molecules	2	2	2
Glycans	4	4	4
Water molecules	945	1633	317
Ions	14	13	8
Other solvent	13	1	0
**Refinement**			
*R*_cryst_	0.211	0.176	0.225
*R*_free_	0.240	0.193	0.248
RMS deviation			
Bond lengths (Å)	0.015	0.022	0.018
Bond angles (°)	1.25	1.65	1.78
Ramachandran (%)			
Favored	97.05	98.4	97.3
Allowed	2.83	1.6	2.7
Outliers	0.12	0	0
Mean *B* factors			
Protein (Å^2^)	34.90	15.52	33.57
Glycans (Å^2^)		20.93	49.26

**Figure 4.**
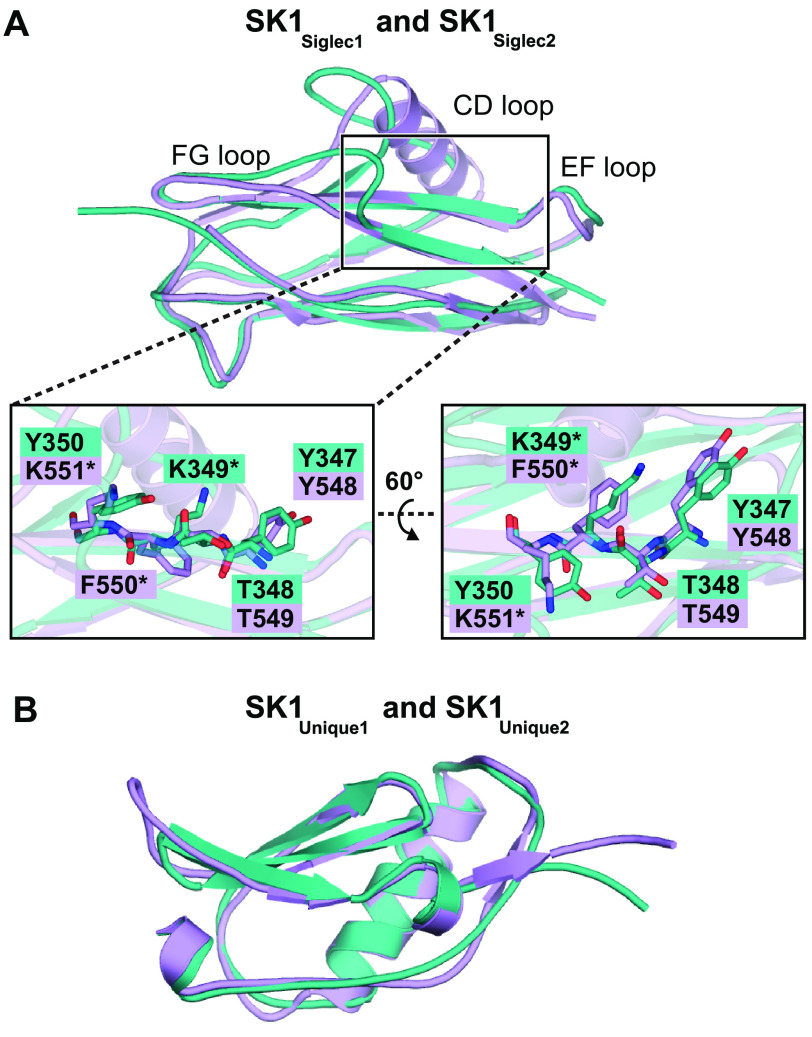
**Structural comparison of the tandem Siglec and Unique domains.** *A*, and *B*, cartoon diagrams of unliganded SK1_BR_. SK1_Siglec1_ and SK1_Unique1_ are colored in *teal*, and the SK1_Siglec2_ and SK1_Unique2_ are colored in *lavender*. *A*, 98 eight atoms were aligned, and 26 were rejected after 5 cycles to output an RMS deviation of 1.058 Å. The noncanonical binding motifs of SK1_Siglec1_ and SK1_Siglec2_ are shown as *sticks*, and residues that deviate from the canonical YTRY motif definition are noted with an *asterisk*. *B*, 72 were aligned, and 3 were rejected after 2 cycles to output an RMS deviation of 0.639 Å.

As is anticipated from the amino acid sequence conservation, the individual Siglec domains and Unique domains exhibit structural similarity, with RMS deviations in Cα position of 1.058 Å between SK1_Siglec1_ and SK1_Siglec2_ and 0.639 Å between SK1_Unique1_ and SK1_Unique2_ (Fig. 4). To evaluate the basis for the higher overall RMS deviations in Cα position of the Siglec domains, we overlaid SK1_Siglec1_ and SK1_Siglec2_ and identified whether this was a global difference or whether the structural difference was localized to specific regions. We identified disproportionately large structural deviations in the CD, EF, and FG selectivity loops that surround the putative sialoglycan-binding pockets (Fig. 4*A*), which contain different numbers of residues, with the FG loop of SK1_Siglec2_ being so short that it is effectively absent. The maximal displacement of these loops in overlays is 10.9 Å for the CD loop, 3.2 Å for the EF loop, and 8.4 Å for the FG loop. In the CD loop, this also manifests as a difference in secondary structure in which the CD loop of SK1_Siglec1_ lacks secondary structure, whereas the CD loop of SK1_Siglec2_ folds into an α-helix. In addition, the CD loop of SK1_Siglec2_ is displaced from the binding pocket when compared with the CD loop of SK1_Siglec1_. Together, these differences in loop length and structure result in a larger and more open binding site as compared with SK1_Siglec1_. This large binding pocket of SK1_Siglec2_ is somewhat reminiscent of the binding pocket in the SrpA adhesin from *S. sanguinis*, which also lacks the FG loop (15–17).

### Structures of S. sanguinis SK1_BR_ bound to sialoglycans

**Figure 5.**
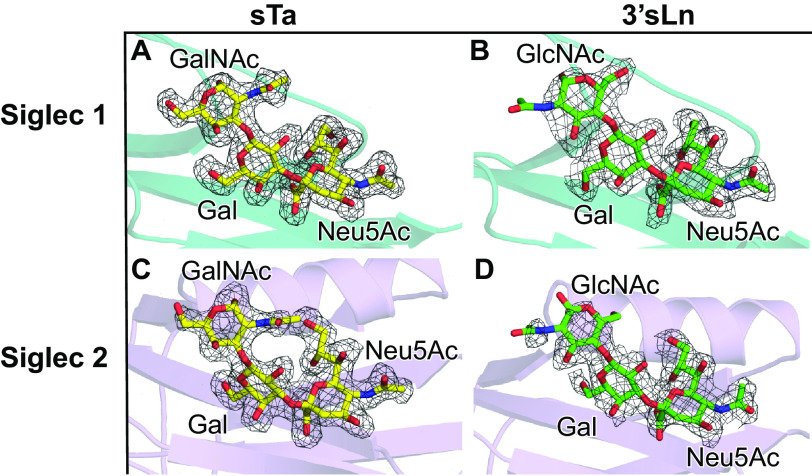
**Ligand electron density.** *A–B*, SK1_Siglec1_ is shown in *teal* cartoon. *C–D*, SK1_Siglec2_ is shown in *lavender* cartoon. *A, C*, sTa and *B, D*, 3′sLn are shown as *yellow* and *green sticks*, respectively. Oxygen atoms are colored in *red*, and nitrogen atoms are in *blue*. Ligands were manually placed in Coot after refinement of the protein and prior to solvent placement. The ligands were then refined with rigid-body and real space refinement in Coot prior to solvent placement and final structure refinements. |*F*_o_| − |*F*_c_| electron density maps were calculated from coordinates that had been refined in Phenix (40) for three rounds after the removal of the sialoglycans from the model. Maps are contoured at 3σ and are shown in *dark gray mesh*.

**Figure 6.**
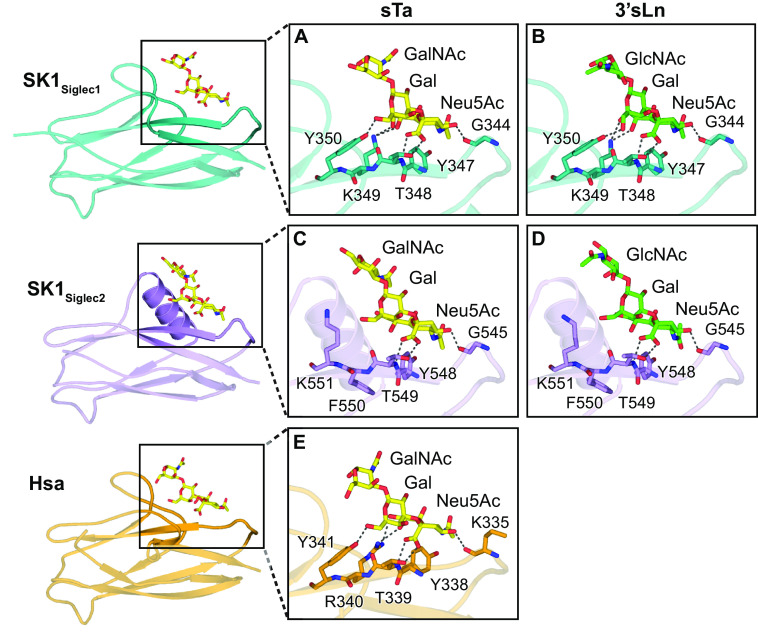
**SK1 interactions with ligands.** The structures of the Siglec domains of *A–B*, SK1_Siglec1+Unique1_, *C–D*, SK1_Siglec2+Unique2_, and *E*, Hsa (PDB entry 6EFD) (18) are shown in cartoon in *teal*, *lavender*, and *orange*, respectively. The adhesin residues that hydrogen bond with the ligands are shown as *sticks*. Hydrogen bonds between the adhesins and ligands are shown as *dark gray dashed lines*. The ligands sTa and 3´sLn are shown as *yellow* and *green sticks*, respectively. Oxygen and nitrogen atoms are colored *red* and *blue*, respectively.

Prior glycan array analysis identified that SK1_BR_ can bind to a broad range of defined, synthetic sialoglycan ligands. It was not clear, however, whether SK1_Siglec1_ and SK1_Siglec2_ are both important for these interactions or how the noncanonical YTRY motifs support glycan binding. We therefore determined cocrystal structures of SK1_BR_ soaked with either 10 mm sialyl T-antigen (sTa), a core 1 glycan that can be conjugated to Ser or Thr residues of glycoproteins, or 10 mm 3´-sialyl-*N*-acetyllactosamine (3´sLn), a trisaccharide that can be a component of larger, branched glycans. For both sTa and 3´sLn, we observed the appearance of unambiguous electron density adjacent to the noncanonical YTRY motif of both SK1_Siglec1_ and SK1_Siglec2_. We were able to model sTa and 3´sLn with confidence into this electron density, and the hydrogen bond networks observed are consistent with specific binding (Figs. 5, *A–D*, and 6, *A–D*). Thus, SK1_Siglec1_ and SK1_Siglec2_ are both capable of binding sialoglycan ligands and can do so simultaneously. Local conformational changes observed upon binding were slight. When each domain is individually aligned to the corresponding unliganded domain, the RMS deviations in the Cα positions is <0.3 Å (Table S1). The biological significance of conformational changes of this magnitude cannot be determined.

As compared with adhesins with canonical YTRY motifs (16, 18, 19), the sialic acid of each trisaccharide interacts with the two noncanonical motifs of SK1 via fewer hydrogen-bonding interactions (Fig. 6, *A–E*). The contacts that are similar between SK1 and structurally characterized adhesins with YTRY motifs include interactions with the backbone of the N-terminal Tyr and side chain of the Thr residues in both noncanonical YTRY motifs (Fig. 6). Hydrogen-bonding to sialic acid additionally occurs via a backbone carbonyl in the EF loop (SK1^G344^ and SK1^G545^) (Fig. 6, *A–D*). Although not previously reported for the SRR adhesins, this interaction is conserved across other adhesins (GspB^I479^, SrpA^R342^, and Hsa^K335^) (Fig. 6*E*) (6, 17, 18).

SK1_Siglec1_ hydrogen-bonds to the sialic acid and galactose of both sTa and 3´sLn via the YTKY sequence (Fig. 6, *A* and *B*). Here, the Arg → Lys substitution eliminates one side-chain hydrogen bond (Fig. 6*A* and Figs. S2*A* and S3*A*), but the overall interaction remains similar to that supported by a canonical YTRY motif, like Hsa from *S. gordonii* strain Challis or GspB from *S. gordonii* strain M99 (Figs. S2*B* and 6*E*) (16).

In contrast, the Phe and Lys residues in the SK1_Siglec2_ YTFK sequence do not form hydrogen-bond contacts with the ligand. In addition, the YTFK sequence of SK1_Siglec2_ does not hydrogen-bond to the galactose or the variable third sugar, GalNAc/GlcNAc, of sTa/3´sLn (Fig. 6, *C* and *D*). Compensating for this, side chains in the helical CD loop make additional hydrogen-bond contacts (Figs. S2, *C* and *D*, and S3, *B* and *C*). Both observations indicate that sialic acid recognition by SK1_Siglec2_ differs from characterized ligand interactions in the GspB, Hsa, and SrpA adhesins (16, 18, 19).

**Figure 7.**
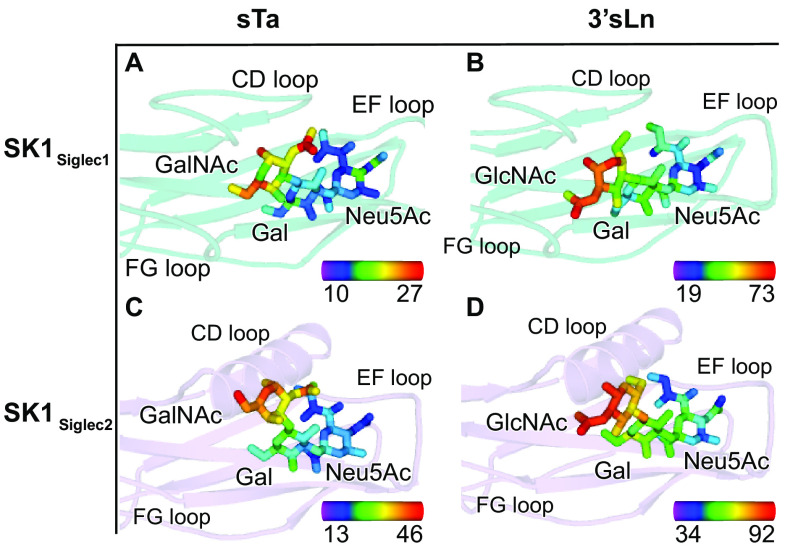
**Temperature factor analysis of Siglec domains and bound ligands.** *A–D*, SK1_Siglec1_ is shown in *teal cartoon*, and SK1_Siglec2_ is shown in *lavender cartoon*. The sTa liganded structures are shown in *A* and *C*, and the 3´sLn liganded structures are shown in *B* and *D*. Both sTa and 3´sLn are shown as *sticks* and colored by temperature factor, where *blue* represents a low temperature factor, and *red* represents a high temperature factor as depicted by the scale in the *bottom right corner* of each panel. The scale values are in Å^2^.

This reduced number of binding contacts is reflected in temperature factor analysis of the ligand. Crystallographic temperature factors can give a rough estimate of inherent mobility Fig. 7 and Fig. S4. It is important to note that the CD, EF, and FG loops, the YTRY motifs, and the ligands do not participate in crystal contacts in any of the three structures; as a result, the temperature factors are not influenced by crystal packing interactions. Here, the low temperature factors of sTa and SK1_Siglec1_ suggest that the ligand has little mobility when bound, which may be interpreted as strong binding (Fig. 7*A*). In contrast, the higher temperature factors of 3´sLn bound to SK1_Siglec1_ or of either sTa or 3´sLn bound to SK1_Siglec2_ suggest higher mobility, which suggests that these could be lower-affinity ligands (Fig. 7, *B–D*). Nevertheless, in all cases, the temperature factor is the lowest at the sialic acid and increases over the length of the trisaccharide, consistent with lower ligand mobility at the sialic acid and increased mobility at the reducing end sugar.

**Figure 8.**
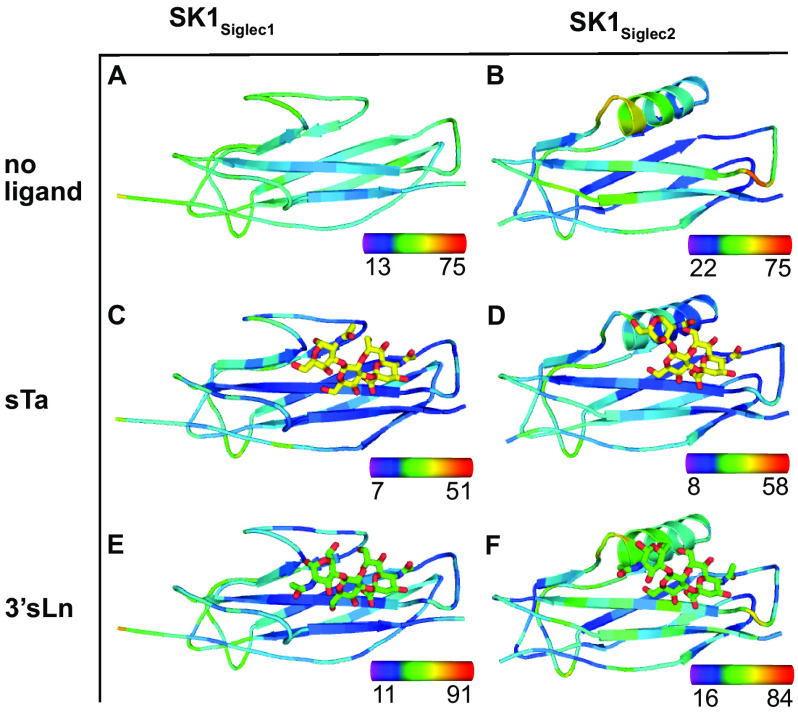
**Siglec domain colored by temperature factor.** *A–B*, SK1 unliganded, (*C–D*) sta-bound, and *E–F* 3′sLn-bound are shown in cartoon and colored by temperature factor, where *blue* represents a low temperature factor, and *red* represents a high temperature factor. The *color bars* in the *bottom right corner* of each panel indicate the ranges of B factors in Å^2^. sTa and 3´sLn are shown as *sticks*. The oxygen and nitrogen atoms are colored *red* and *blue*, respectively.

We then performed the converse analysis, assessing the temperature factors of each Siglec domain near the ligand (Fig. 8). In the unliganded state, we observe that the selectivity loops have elevated temperature factor values as compared with the V-set Ig fold that forms the core of the Siglec domain, suggesting that they have increased mobility (Fig. 8, *A* and *B*). Upon ligand binding, the temperature factors of all loops become more similar to the temperature factors of the Siglec domain core in both SK1_Siglec1_ and SK1_Siglec2_ (Fig. 8, compare *A* and *B* with *C–F*). Moreover, the temperature factors of the YTKY motif in SK1_Siglec1_ decreased upon ligand binding (Fig. 8, compare *A*, *C*, and *E*). The temperature factors of the YTFK motif in SK1_Siglec2_ decrease upon sTa binding but do not decrease upon 3´sLn binding (compare Fig. 8, compare *B*, *D*, and *F*). This stabilization is more pronounced in the sTa-bound SK1_Siglec2_ than in the 3´sLn-bound SK1_Siglec2_, possibly because of the additional hydrogen bond between sTa and the CD helix. This extra hydrogen bond further links the YTRY region and the CD helix, decreasing the mobility of both regions (Fig. S2, *C* and *D*). Together, this analysis suggests that the binding of ligands stabilizes the positions of the selectivity loops in both domains. This is consistent with prior molecular dynamics simulations of the Hsa adhesin from *S. gordonii* strain Challis, which suggested that these selectivity loops can adjust to optimize the interaction to ligands (18).

**Figure 9.**
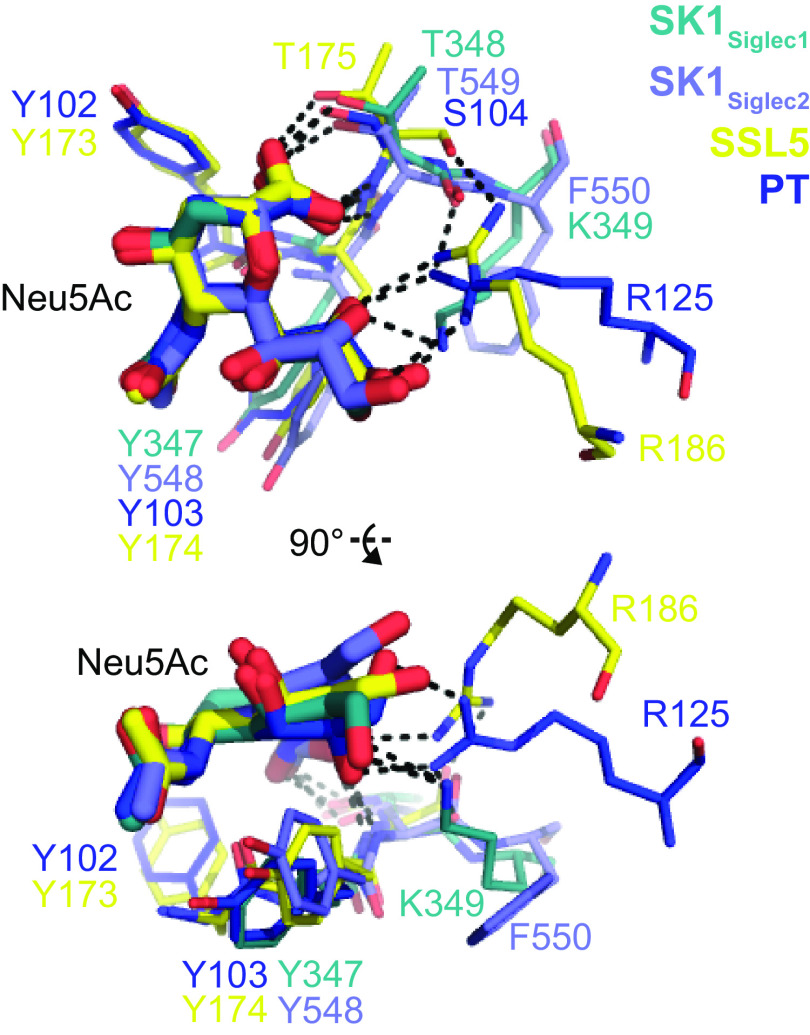
**Comparison of bacterial sialic acid–binding pockets.** Shown here is an overlay of the two Siglec domains of SK1_BR_ with staphylococcal superantigen-like protein 5 (*SSL5*; PDB entry 2R61) and pertussis toxin (*PT*; PDB entry 1PTO) (48), two proteins in which the sialic acid recognition motif has previously been identified (22).

Intriguingly, the interactions between sialic acid of sTa or 3´sLn and the YTRY motif contain structural parallels to staphylococcal superantigen-like protein SSL5 bound to sialyl Lewis^X^ (Fig. 9) (22). Prior comparisons of SSL5 with a range of evolutionarily unrelated sialic acid–binding proteins suggested a common sialic acid recognition motif that contains two features: 1) a YY(T/S) motif on an edge strand of a β sheet and 2) an arginine distant in sequence but spatially adjacent to the YY(T/S) motif (22). Despite the somewhat different sequence elements in the sialic acid–binding motif of SK1_BR_ and other SRR adhesins, the hydrogen-bonding pattern to the sialic acid is similar to that of SSL5 (Fig. 9). The arginine, or lysine in the case of SK1_Siglec1_, associated with sialic acid binding is provided from within the YTRY motif of SK1_Siglec1_ (Lys^349^) and other Siglec-like adhesins, whereas it is outside of the YY(T/S) motif in SSL5 (Arg^186^) and PT (Arg^125^) and other sialic acid–binding proteins. The interactions between the binding pocket and sialic acid may be a product of convergent evolution (22).

### SK1_Siglec1_ and SK1_Siglec2_ have unique selectivity profiles and exhibit synergistic binding

Based upon our observation that both SK1_Siglec1_ and SK1_Siglec2_ bind sialoglycans in a cocrystal structure, we tested the relevance of these interactions in binding to defined, synthetic glycans. To do this, we developed recombinant GSH S-transferase (GST)–tagged proteins (Fig. S5) containing either the first Siglec and Unique domains, SK1_Siglec1+Unique1_ (SK1^252–455^), or the second Siglec and Unique domains, SK1_Siglec2+Unique2_ (SK1^449–660^) and compared the binding of these isolated binding modules to that of full-length SK1_BR_.

**Figure 10.**
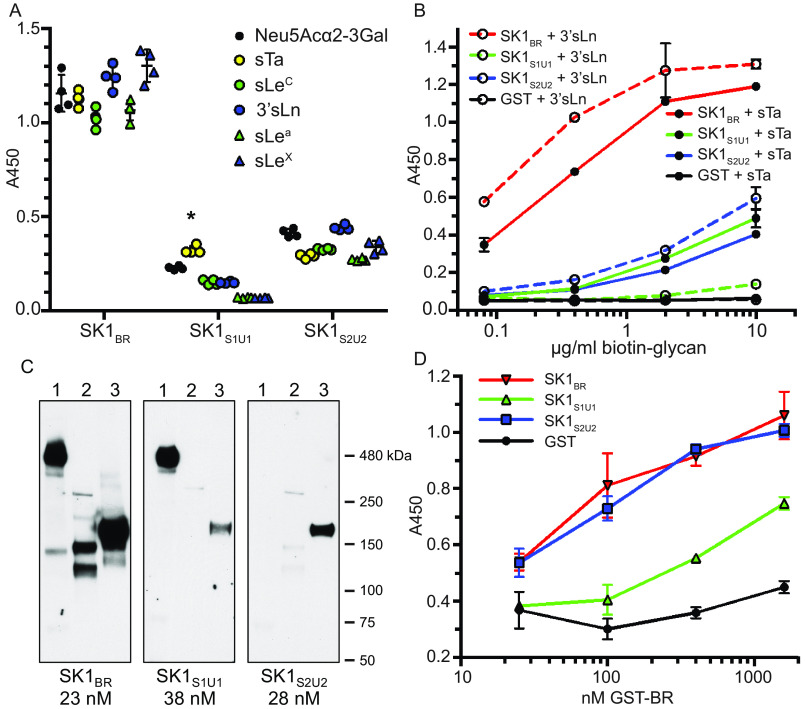
**Binding of SK1_BR_ and split variants to glycans and glycoproteins.** *A*, biotin-glycan binding to immobilized GST-tagged SK1_BR_ and the split binding modules (*n* = 4 technical replicates). The *asterisk* indicates binding that was significantly greater than the level of binding to all other glycans in the set of six (*p* < 0.05 using a two-way analysis of variance with Tukey's correction for multiple comparisons). *B*, binding of biotinylayed sTa or 3´sLn to immobilized GST-tagged SK1_BR_ and split constructs (*n* = 3 technical replicates). *C*, binding of GST-tagged SK1_BR_ and split constructs to glycoproteins in human plasma (*lane 1*), platelet lysate (*lane 2*), or submandibular sublingual saliva (*lane 3*). *D*, binding of GST-tagged SK1_BR_ and SK1_BR_ deletion constructs to immobilized human platelets (*n* = 3 technical replicates). In *A*, *B*, and *D*, mean values ± standard deviation are indicated. In cases where *error bars* are not evident, the deviations were smaller than the size of the symbol used for the data point. Background values for GST alone were not subtracted but are shown in *B* and *D*.

We began by evaluating how each repeat bound to a small library of tri- and tetrasaccharide sialoglycans at a single concentration of ligand (Fig. 10*A*). Both SK1_Siglec1+Unique1_ and SK1_Siglec2+Unique2_ bound to at least some of the tested sialoglycans, albeit less strongly than did full-length SK1_BR_. Consistent with the crystallographic temperature factor analysis (Fig. 7), SK1_Siglec1+Unique1_ showed a statistically significant preference for sTa. In contrast, SK1_Siglec2+Unique2_ appears to be more broadly selective. The latter finding is consistent with the observation that the SK1_Siglec2_ domain does not make hydrogen-bonding contacts to the second and third sugars of trisaccharide in the crystal structures (Fig. 6, *C* and *D*).

We then performed a more detailed dose-dependent binding to sTa and 3´sLn (Fig. 10*B*). This analysis indicates that both SK1_Siglec1+Unique1_ and SK1_Siglec2+Unique2_ bind to sTa ∼100-fold less strongly than the full-length SK1_BR_, which contains the two subdomains in tandem. This suggests the possibility of binding synergy when in the presence of high concentrations of carbohydrate. Physiologically, small portions of large and complex branched glycans could have a similar appearance as high local concentrations in a binding assay. Such high levels of a particular glycan are hypothesized for glycoproteins that contain oriented glycan patches (23).

### Synergistic properties of SK1_BR_ affect binding to host receptors

We next evaluated whether binding to the synthetic glycans explains how SK1_BR_ interacts with host glycoproteins. Previously identified ligands for the Siglec-like SRR adhesins are consistent with their biological roles in oral commensalism and the pathogenesis of infective endocarditis. These include salivary MUC7, platelet GPIbα, and several *O*-glycosylated plasma glycoproteins. We therefore evaluated the interactions of GST-tagged SK1_BR,_ SK1_Siglec1+Unique1_, and SK1_Siglec2+Unique2_ with human salivary, platelet, and plasma glycoprotein targets via Far Western blotting. Isolated binding regions SK1_Siglec1+Unique1_ and SK1_Siglec2+Unique2_ both bound modestly to MUC7 compared with binding by the tandem domains of SK1_BR_ (Fig. 10*C*). SK1_Siglec1+Unique1_ readily bound a 460-kDa plasma protein, whereas SK1_Siglec2+Unique2_ did not. Here, the tandem domains of SK1_BR_ did not increase the binding over what was observed for SK1_Siglec1+Unique1_. Neither SK1_Siglec1+Unique1_ nor SK1_Siglec2+Unique2_ bound appreciably to GPIbα in the platelet lysate.

We also assessed binding to fixed, immobilized platelets (Fig. 10*D*). SK1_Siglec1+Unique1_ bound weakly, whereas SK1_Siglec2+Unique2_ bound more strongly. There was not a cooperative effect of linking these domains, because the binding of SK1_BR_ to platelets could be fully explained by the binding of SK1_Siglec2+Unique2_. These results suggest that there is a high-affinity ligand for SK1_BR_ on intact platelets that is due primarily to binding by SK1_Siglec2+Unique2_. The combined results indicate that SK1_BR_ can bind multiple simple and complex sialoglycan ligands on biological targets via a combination of interactions.

## Discussion

All previously determined structures of the binding regions of Siglec-like adhesins have a single Siglec domain and a single Unique domain. This prior work has shown that sialic acid–binding affinity largely stems from binding to a YTRY motif with the selectivity tuned via adjacent loop regions of the Siglec domain (18). Our data are consistent with each repeat of SK1_BR_ following the same principles for binding and selectivity. Specifically, the sialoglycan binds via specific interactions between sialic acid and the noncanonical YTRY motif. Given this finding in conjunction with studies of SrpA, the YTRY motif can be more formally defined as ϕTR*X*, where ϕ represents Trp, Phe, or Tyr, and *X* represents Tyr, Thr, Glu, His, or Lys (Fig. 2) (17, 19). This ϕTR*X* motif represents a sequence modification of the YY(S/T) motif found in other sialic acid–binding proteins, with ϕT of the **ϕ**TR*X* corresponding to the YT of Y**Y**(S/**T**). As a result, both sequence motifs interact with sialic acid via a similar pattern of hydrogen bonds. For the Siglec-like adhesins, the selectivity loops may control the identity of the preferred sialoglycan. In SK1_Siglec1_ and SK1_Siglec2_, these selectivity loops differentially impact the size of the binding pocket and the orientation of the ligand, resulting in unique selectivity profiles.

**Figure 11.**
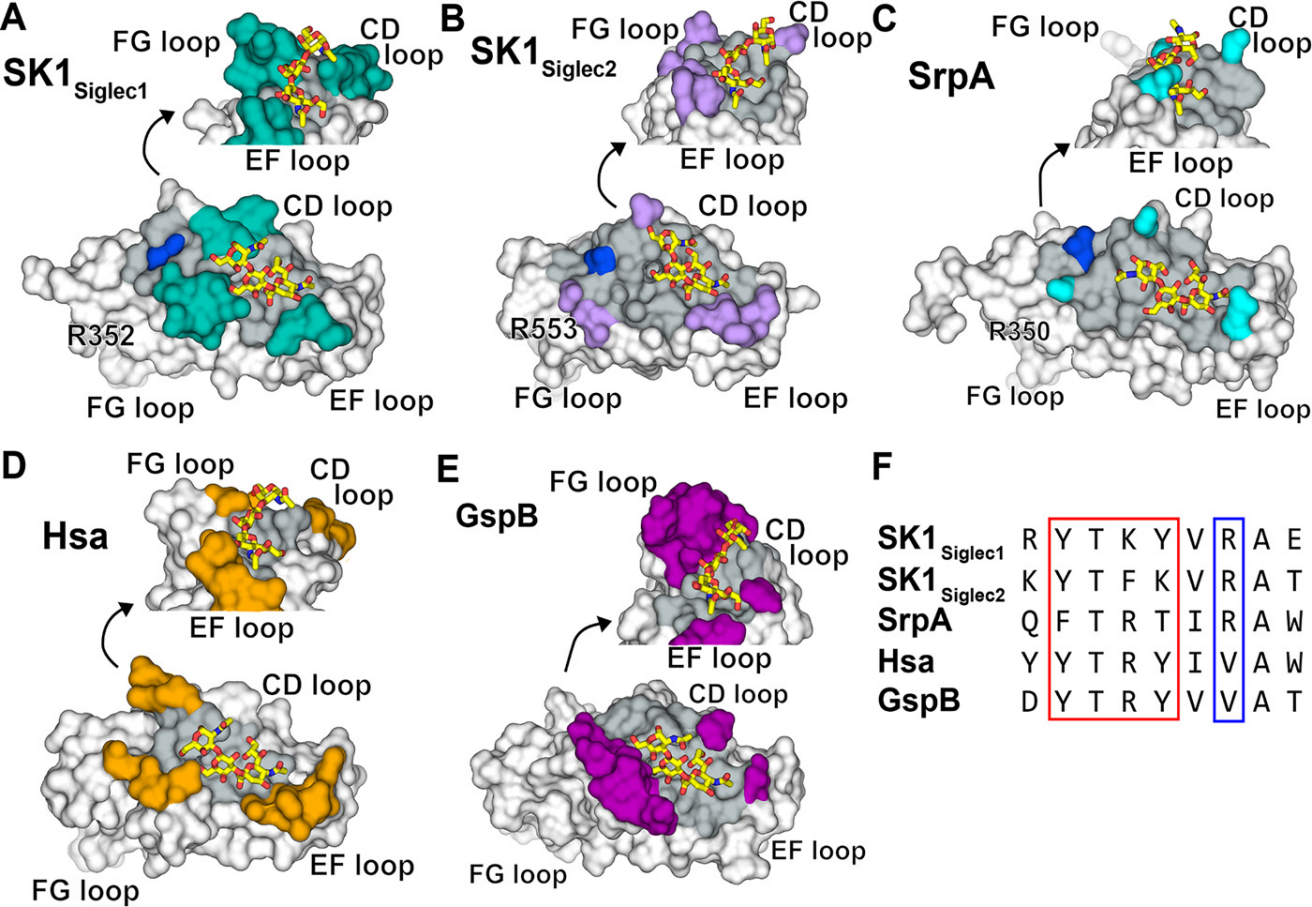
**SRR adhesins binding pocket size comparison.** *A–E*, the adhesins are shown in surface representations (16–18). The top image of the binding site is rotated 80° around the *z* axis and 70° around the *x* axis. The binding pocket of each is colored in *gray*, and the portions of the CD, EF, and FG loops that create the walls of the binding pocket are colored in *teal*, *lavender*, *cyan*, *orange*, and *magenta* for SK1_Siglec1_, SK1_Siglec2_, SrpA, Hsa, and GspB, respectively. The Arg distal to the YTRY motif is colored *blue*. Sialyl T antigen is shown as *yellow sticks* bound to each adhesin. *F*, multiple sequence alignment of the above adhesins is shown. The YTRY motif is outlined in *red*, and the distal Arg is outlined in *blue*.

**Figure 12.**
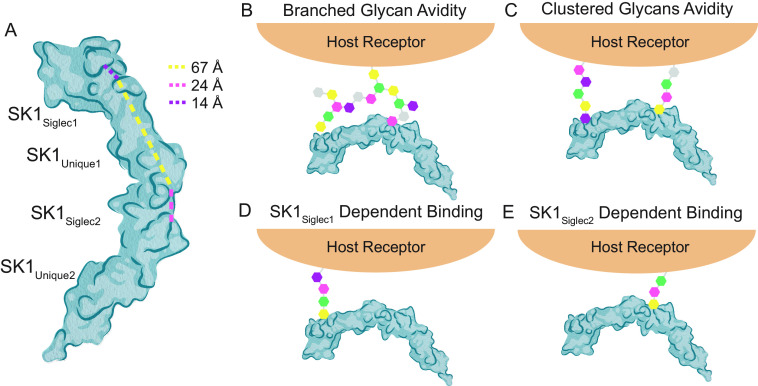
**Model of target-specific effects of SK1.** *A*, the distance between the two binding sites is 67 Å, measured from the C_α_ atoms of Tyr-347, the first residue in the YTRY motif of SK1_Siglec1_, and Arg-553, the distal arginine residue of SK1_Siglec2_. The length of the binging site in SK1_Siglec1_ is 14 Å, measured from the Cα atoms of Gly-344 and Lys-349. The length of the binding site in SK1_Siglec2_ is 24 Å, measured from the Cα atoms of Gly-545 to Arg-553. *B–E*, note that the glycans shown here are only meant to serve as a hypothetical glycan structure and are not meant to represent a specific, defined glycan target. *B*, multivalent binding of a branched glycan. Given the distance between the two binding sites, SK1_BR_ could bind the same branched glycan with both SK1_Siglec1_ and SK1_Siglec2_. *C*, multivalent binding of a patch of clustered glycans. Each binding site of SK1_BR_ binds a separate glycan structure. *D*, SK1_Siglec1_ dominant binding. *E*, SK1_Siglec2_ dominant binding. Given the openness of the binding site and the presence of the distal Arg, SK1_Siglec2_ may bind a short saccharide, two short saccharides, or a longer hexasaccharide.

Although the ϕTR*X* motif and selectivity loops of SK1_BR_ support our current understanding of sialoglycan binding, two unique features highlight unanswered questions regarding the link between sialoglycan binding and adhesion. For instance, SK1_Siglec1+Unique1_ exhibited selectivity for sTa but had a lower affinity for glycoproteins than SK1_Siglec2+Unique2_. In contrast, SK1_Siglec2+Unique2_ binds well to host components but poorly to purified trisaccharides. It is possible that the trisaccharides tested do not include the full biological ligand. SrpA similarly has a larger and more open binding pocket that could possibly accommodate a larger ligand (Fig. 11*C*). Hypotheses for the biological ligand can be developed by considering parallels to the SrpA adhesin from *S. sanguinis* strain SK36. Like SK1_Siglec2+Unique2_, SrpA binds poorly to purified tri- and tetrasaccharides *in vitro*, but SrpA binds robustly to human platelets. Lacking an FG loop, SrpA has a significantly larger binding site than many other SRR adhesins. This increased size of the binding site opens to a distal arginine residue (Fig. 11, *C* and *F*). For this reason, it has been proposed that physiological targets of SrpA may include a disialylated hexasaccharide or a patch of multiple, oriented glycans (17). Either possibility for the ligand could promote cooperativity that would be expected to increase adhesion to host targets. Like SrpA, SK1_Siglec2_ similarly has a large sialoglycan-binding site because of the small size of the FG loop (Figs. 11*B* and 12*A*). We propose that SK1_Siglec2_ may therefore also bind a “core 2” sialoglycan or patch of oriented glycans (Fig. 12, *b* and *c*). In contrast, the distal arginine residue in SK1_Siglec1_ is occluded from the sialoglycan-binding site by a larger FG loop, likely prohibiting interactions with a sialoglycan longer than a trisaccharide (Figs. 11*A* and 12*A*); however, binding of multiple glycans is still a possibility. Alternatively, a large binding site alone may be sufficient for some adhesins to bind larger saccharides. Hsa also has a large and open binding site but with no distal arginine (Fig. 11, *D* and *F*). This is consistent with the ability of Hsa to bind both trisaccharides and larger, branched sialoglycans (2, 6, 24).

SK1_BR_ also demonstrates how individual binding domains *versus* tandem linkage can differentially contribute to the affinity for host targets. Conceptually, tandem linking of these two binding regions would be expected to confer cooperative binding capability in binding to some biological targets (25). Or to put it another way, if a target protein contains glycan modifications that are correctly spaced and oriented, this adhesin could bind more strongly to host receptors via multivalent binding (Fig. 12, *B* and *C*) (26). Synergy was indeed observed between SK1_Siglec1+Unique1_ and SK1_Siglec2+Unique2_ when binding to platelet lysate or to salivary glycoproteins (Fig. 10*C*). This suggests that both binding regions contribute to adherence for certain host targets and could be explained by binding to either a large branched glycan structure or a patch of clustered glycans (Fig. 12, *B* and *C*).

On the other hand, binding of SK1_Siglec1+Unique1_ to human plasma is roughly equivalent to that of SK1_BR_, suggesting that SK1_Siglec1+Unique1_ has a high-affinity sialoglycan target in human plasma, but SK1_Siglec2+Unique2_ does not. SK1_Siglec1+Unique1_ may be responsible for adherence to human plasma, consistent with an SK1_Siglec1_-dependent binding model (Figs. 10*C* and 12*D*). SK1_Siglec2+Unique2_ seems to be solely responsible for adherence to immobilized platelets, suggesting an SK1_Siglec2_-dependent binding mode (Figs. 10*D* and 12*E*). The use of tandem repeats and multivalent binding capabilities could confer two distinct evolutionary advantages. First, tandem repeats can be separately mutated for a faster evolution. For example, if an adhesin contains two binding regions following a gene duplication event (27), the domains could then be individually mutated, with each domain conferring different selectivity for host receptors. Divergent evolution of the two binding domains could effectively double the evolutionary rate, leading to faster adaptation. Tandem linkage of binding domain modules could also allow an individual binding domain to evolve through an intermediate with lower affinity and broader specificity. This could allow for the evolution of larger changes in selectivity (28–30).

Second, the combined action of two binding domains could allow binding of this adhesin to a broader range of targets. Linking an sTa-specific domain with a domain of another or broader selectivity could allow adherence to either platelets or to other host targets. Increasing the range of ligands an adhesin can bind could increase tropism and allow bacteria to migrate from one tissue to another. Avidity and affinity optimization of protein scaffolds for recognition of on- and off-target biomolecules can increase the specificity of cellular targeting (31). This heteromultivalent binding could be important for increasing specificity for a target tissue (26, 32, 33).

Taken together, the findings reported here get us closer to addressing unanswered questions in the field. The finding that noncanonical motifs of SRR adhesins interact robustly with sialoglycan ligands reveals them as important for host interaction. More importantly, these results provide insight into how adhesive proteins adapt to various biological niches with different host receptors and provide evidence for adhesion to patches of oriented glycans (24). This feeds into ongoing work that seeks to develop a predictive model for streptococcal pathogenicity.

## Experimental procedures

### Expression and purification of SK1_BR_

DNA encoding residues 252–660 of the full-length SK1 adhesins, termed SK1_BR_, was cloned into the pBG101 vector (Vanderbilt), which encodes an N-terminal His_6_-GST affinity tag followed by a cleavage sequence for the 3C precision protease. SK1_BR_ was expressed in *Escherichia coli* BL21(DE3) cells. The cells were grown in LB at 37 °C to an *A*_600 nm_ of ∼0.6 and expression induced for 4 h with 0.5 mm isopropyl β-d-thiogalactopyranoside. The cells were harvested by centrifugation at 9220 × *g* for 15 min. The pellets were resuspended in 250 ml of lysing buffer (20 mm Tris-HCl, 150 mm NaCl, pH 7.6) supplemented with 2 µg/ml pepstatin, 2 µg/ml leupeptin, 1 µg/ml DNase, and 1 mg/ml lysozyme. The cells were lysed by sonication. The lysate was clarified by centrifugation at 38,465 × *g* for 1 h, and then the supernatant was filtered (0.45 μm) and loaded onto a 5-ml His Trap column. SK1_BR_ was eluted with 75 mm imidazole elution buffer (20 mm Tris-HCl, 150 mm NaCl, 75 mm imidazole, pH 7.6). The N-terminal His_6_-GST tag was cleaved with 3C precision protease (2 mg/ml SK1_BR_, 20 mm Tris, 150 mm NaCl, 75 mm imidazole, pH 7.6). The imidazole was diluted, and the protein was concentrated using a 30-kDa molecular-weight-cutoff centrifugal concentrator. Concentrated protein was passed through a His Trap column to remove the cleaved His_6_-GST tag. The protein was then purified by Superdex 200 size exclusion column (20 mm Tris-HCl, pH 7.6, 150 mm NaCl). Bradford assay was used to determine the final protein concentration.

### Synthesis of sTa trisaccharide Neu5Acα2–3Galβ1–3GalNAc

Galβ1–3GalNAc (34) (30 mg, 0.078 mmol), Neu5Ac (37 mg, 0.117 mmol), and cytidine 5′-triphosphate (CTP) (66 mg, 0.117 mmol) were dissolved in a solution containing Tris-HCl buffer (8.0 ml, 100 mm, pH 8.5) and MgCl_2_ (20 mm). *Neisseria meningitidi*s cytidine 5′-monophosphate-sialic acid (CMP-sialic acid) synthetase (35) (1.0 mg) and *Pasteurella multocida* sialyltransferase 1 M144D (36) (1.5 mg) were then added. The reaction was carried out by incubating the reaction mixture in an incubator shaker at 37 °C for 12 h. The reaction was monitored by TLC (EtOAc/MeOH/H_2_O/HOAc = 4:2:1:0.1, by volume) with *p*-anisaldehyde sugar staining and MS. When an optimal yield was achieved, the same volume (8 ml) of prechilled ethanol was added, and the mixture was incubation at 4 °C for 30 min. The sample was centrifuged, and the precipitates were removed. The supernatant was concentrated, passed through a BioGel P-2 gel filtration column, and eluted with water to obtain the partially purified product. Further purification was achieved using silica gel chromatography (EtOAc/MeOH/H_2_O = 4:2:1, by volume) and a final pass through of a P-2 gel filtration column to produce pure sTa (47 mg, 86%). NMR data were in agreement with those reported previously (37).

### Crystallization and structure determination

SK1_BR_ (72 mg/ml in 150 mm NaCl, 20 mm Tris, pH 7.6) was crystallized using the hanging-drop vapor-diffusion method at 25 °C using a reservoir solution containing 20% (w/v) PEG 3350, 0.2 m MgSO_4_, 0.01 m SrCl_2_. The crystals were harvested 1 week later. To obtain the sialoglycan-bound SK1_BR_ structures, crystals of the unliganded SK1_BR_ were grown for 1 week, removed from the mother liquor, and placed in a new drop containing all of the crystallization components (20% (w/v) PEG 3350, 0.2 m MgSO_4_, 0.01 m SrCl_2_) and either 20 mm sTa (synthesized in-house) or 20 mm 3´sLn (Glycotech). The crystals were allowed to incubate with the glycan for 1 h at room temperature before harvesting.

The crystals were cryoprotected with 40% (1:1 ethylene glycol:glycerol) and 60% reservoir solution and then cryocooled by plunging in liquid nitrogen. X-ray diffraction data were collected using the Advanced Photon Source Beamline 21-ID-F and a Rayonix MX300 detector. The data were processed using HKL2000 (38). The data collection statistics are in Table 1.

The structure of unliganded SK1_BR_ was determined by molecular replacement using the Phaser (39) subroutine in Phenix (40) and the coordinates of the binding region of the unliganded adhesin from *S. mitis* strain unliganded NCTC10712 (PDB entry 6EFF) (18) as the search model. All solvent molecules were removed from the coordinates prior to searches. Molecular replacement required two separate steps. The first step used the unliganded Siglec domain (residues 244–370) as the search model and identified four copies of the Siglec domain. The coordinates for the Siglec domain were then fixed, and a second step using the Unique domain as the search model (residues 371–445) identified three copies of the Unique domain. The final Unique domain was manually placed, and the connections between domains were made during refinement.

Structures of the sialoglycan-bound SK1_BR_ were determined by rigid body refinement of the individual domains of the unliganded SK1_BR_ with all solvent molecules removed. Unambiguous electron density for each sialoglycan was visible in the initial maps. The sialoglycans were manually placed into the difference electron density in Coot (41) immediately following structure determination and prior to solvent placement. The ligands were then individually adjusted in Coot (41) prior to refinement in Phenix (40). To avoid model bias in analysis of the ligand electron density, the ligands were deleted, and the final solvated model was refined in Phenix to produce |*F*_o_| − |*F*_c_| and 2|*F*_o_| − |*F*_c_| electron density maps. The |*F*_o_| − |*F*_c_| maps obtained from this protocol are shown in Fig. 5 at 3σ. Ligand placements were validated with MotiveValidator (42).

The models were improved using real space refinement in Coot (41) and reciprocal space refinement in Phenix (40). For unliganded SK1_BR_, 5% of the reflections (totaling 3466 reflections) were randomly selected to use as the free-R and were held separately for the duration of the refinement. For the liganded structures, the equivalent reflections were selected for use as the free-R in the reflection editor subroutine in Phenix (40). The final models of liganded and sialoglycan-bound structures of SK1_BR_ each contain two copies of SK1_BR_ in each asymmetric unit, with a single copy containing all residues of the purified protein, *i.e.* residues 252–660 of the full-length adhesin. For the sialoglycan-bound structures, one trisaccharide is bound to each Siglec domain, such that a single copy of SK1_BR_ binds two glycans and there are four trisaccharides per asymmetric unit. Refinement statistics and information regarding the content of the models can be found in Table 2.

### Structural analysis

SK1_Siglec1_ (residues 252–377) and SK1_Siglec2_ (residues 455–573) were aligned using PyMOL (43) which rejected 26 atoms from the alignment and calculated an RMS deviation for the remaining Cα positions of 1.058 Å. SK1_Unique1_ (residues 378–454) and SK1_Unique2_ (residues 574–660) were aligned using the same method and had a calculated RMS deviation of 0.639 Å. The maximum distances between the loops of SK1_Siglec1_ and SK1_Siglec2_ were measured from the Cα atoms of SK1^N229^ and SK1^A497^ for the CD loop, SK1^G342^ and SK1^G543^ for the EF loop, and SK1^D365^ and SK1^K565^ for the FG loop. The sequence logo (Fig. 1) was generated using WebLogo 3.7.4 (44) from a Clustal Omega (45) multiple sequence alignment. Ligand–protein interactions were analyzed by PDBsumgenerate (46) and LigPlot (47).

### Adhesins containing individual single unique binding module

Individual binding modules of SK1_BR_, *i.e.* SK1_Siglec1+Unique1_ (residues 252–455) and SK1_Siglec2+Unique2_ (residues 449–660) were designed based upon the manual evaluation of the end of each folded domain in the crystal structure (Fig. S5). SK1_Siglec1+Unique1_ and SK1_Siglec2+Unique2_ were each expressed from pGEX-3X containing an N-terminal GST tag using the protocol detailed above for the full-length SK1_BR_.

### Far Western blotting

Human blood samples were collected under protocol 11-06207, approved by the University of California San Francisco Institutional Review Board, and these studies and protocols abide by the Declaration of Helsinki principles. The binding of GST-tagged SK1_BR_, GST-tagged SK1_Siglec1+Unique1_, and GST-tagged SK1_Siglec2+Unique2_ to plasma, platelet, and salivary glycoproteins was performed as described (6, 15).

### Binding to immobilized platelets

Human blood samples were collected under protocol 11-06207, approved by the University of California San Francisco Institutional Review Board, and these studies and protocols abide by the Declaration of Helsinki principles. Binding to formalin-fixed human platelet monolayers was performed as described (6). In brief, human platelets were freshly prepared, washed, and immobilized in 96-well plates. After blocking nonspecific binding with 1× blocking reagent (Roche) in Dulbecco's PBS (DPBS), the blocking solution was replaced with 50 μl of GST-tagged SK1_BR_, and split binding module proteins were diluted to the indicated concentrations into 1× blocking solution, and the plates were incubated for 1 h at room temperature (∼22°C) with vigorous rocking. The wells were washed three times with 100 µl DPBS, and the bound GST-tagged proteins were detected by using a rabbit anti-GST (Life Technologies) diluted 1:500 in 1× blocking solution, followed by a peroxidase-conjugated goat anti-rabbit (Sigma) diluted 1:5000 in DPBS, along with the chromogenic substrate *o*-phenylenediamine dihydrochloride (Sigma).

### Binding of biotin-glycans to immobilized binding regions

The binding of polyvalent biotinylated glycans (Glycotech) to GST-tagged SK1_BR_ and split binding module proteins immobilized in 96-well plates was performed as described (6). In brief, the wells were coated with the GST-tagged proteins (500 nm in DPBS) by incubating overnight at 4 °C. The wells were washed twice with DPBS, and biotinylated glycans that had been diluted to the indicated concentrations in 1× blocking reagent (Roche) in DPBS were added. The plates were incubated for 90 min at room temperature (∼22°C) with vigorous rocking, and the unbound glycans were removed by washing three times with DPBS. The bound glycans were detected by using peroxidase-conjugated streptavidin (Sigma), followed by the chromogenic substrate *o*-phenylenediamine dihydrochloride (Sigma).

## Data availability

The coordinates and structure factors have been deposited in the RCSB Protein Data Bank with the accession codes 6VS7 (unliganded), 6VT2 (sTa-bound), and 6VU6 (3´sLn-bound). Raw X-ray diffraction data have been deposited with SBGrid with accession codes 754 (sTa-bound), 755 (unliganded), and 756 (3´sLn-bound). The raw data for the binding analyses in Fig. 10 are available upon request from paul.sullam@ucsf.edu. All other data are contained within the article.

## Supplementary Material

Supporting Information
