# Supplementary material for: Tandem sialoglycan-binding modules in a Streptococcus sanguinis serine-rich repeat adhesin create target dependent avidity effects
Source: J Biol Chem. 2020 Aug 20;295(43):14737–49. doi: 10.1074/jbc.RA120.014177 (PMC7586212; doi:10.1074/jbc.RA120.014177)
Supplement: Supporting Information [file supp_295_43_14737__index.html]

Tandem sialoglycan-binding modules in a Streptococcus sanguinis serine-rich repeat adhesin create target dependent avidity effects — Structure of S. sanguinis SK1 adhesin — Supporting Information 

# Tandem sialoglycan-binding modules in a *Streptococcus sanguinis* serine-rich repeat adhesin create target dependent avidity effects

## Supporting Information

- Supporting Information (to be published online) - Supporting tables and figures
