# Supplementary material for: Tandem sialoglycan-binding modules in a Streptococcus sanguinis serine-rich repeat adhesin create target dependent avidity effects
Source: J Biol Chem. 2020 Aug 20;295(43):14737–49. doi: 10.1074/jbc.RA120.014177 (PMC7586212; doi:10.1074/jbc.RA120.014177)
Supplement: Supporting Information [file supp_RA120.014177_160563_1_supp_572213_q77s17.pdf]

Supporting Information

Tandem sialoglycan-binding modules in a *Streptococcus sanguinis* serine-rich repeat adhesin create target dependent avidity effects

Haley E. Stubbs[1], Barbara A. Bensing[2], Izumi Yamakawa[3], Pankaj Sharma[3], Hai Yu[4], Xi Chen[4], Paul M. Sullam[2], T.M. Iverson[3,5,6]*

From the [1]Graduate Program in Chemical and Physical Biology, Vanderbilt University, Nashville, Tennessee; [2]Department of Medicine, Veterans Affairs Medical Center and University of California, San Francisco, California; [3]Department of Pharmacology, Vanderbilt University, Nashville, Tennessee; [4]Department of Chemistry, University of California, Davis, California; [5]Department of Biochemistry, Vanderbilt University, Nashville, Tennessee; [6]Center for Structural Biology, Vanderbilt University, Nashville, Tennessee

*To whom correspondence should be addressed: T.M. Iverson, email: tina.iverson@vanderbilt.edu

Running Title: *S. sanguinis* SK1 adhesin

**RMS deviation for Cα atoms of liganded and unliganded domains**		
	**sTa**	**3'sLn**
**SK1$_{Siglec1}$**	0.237 Å	0.252 Å
**SK1$_{Unique1}$**	0.214 Å	0.196 Å
**SK1$_{Siglec2}$**	0.194 Å	0.191 Å
**SK1$_{Unique2}$**	0.232 Å	0.160 Å

Supplementary Table 1 RMS deviation values for liganded and unliganded domain alignments. Each Siglec and Unique domain from the liganded structures were individually aligned to the same domain in the unliganded structure in Pymol (43).

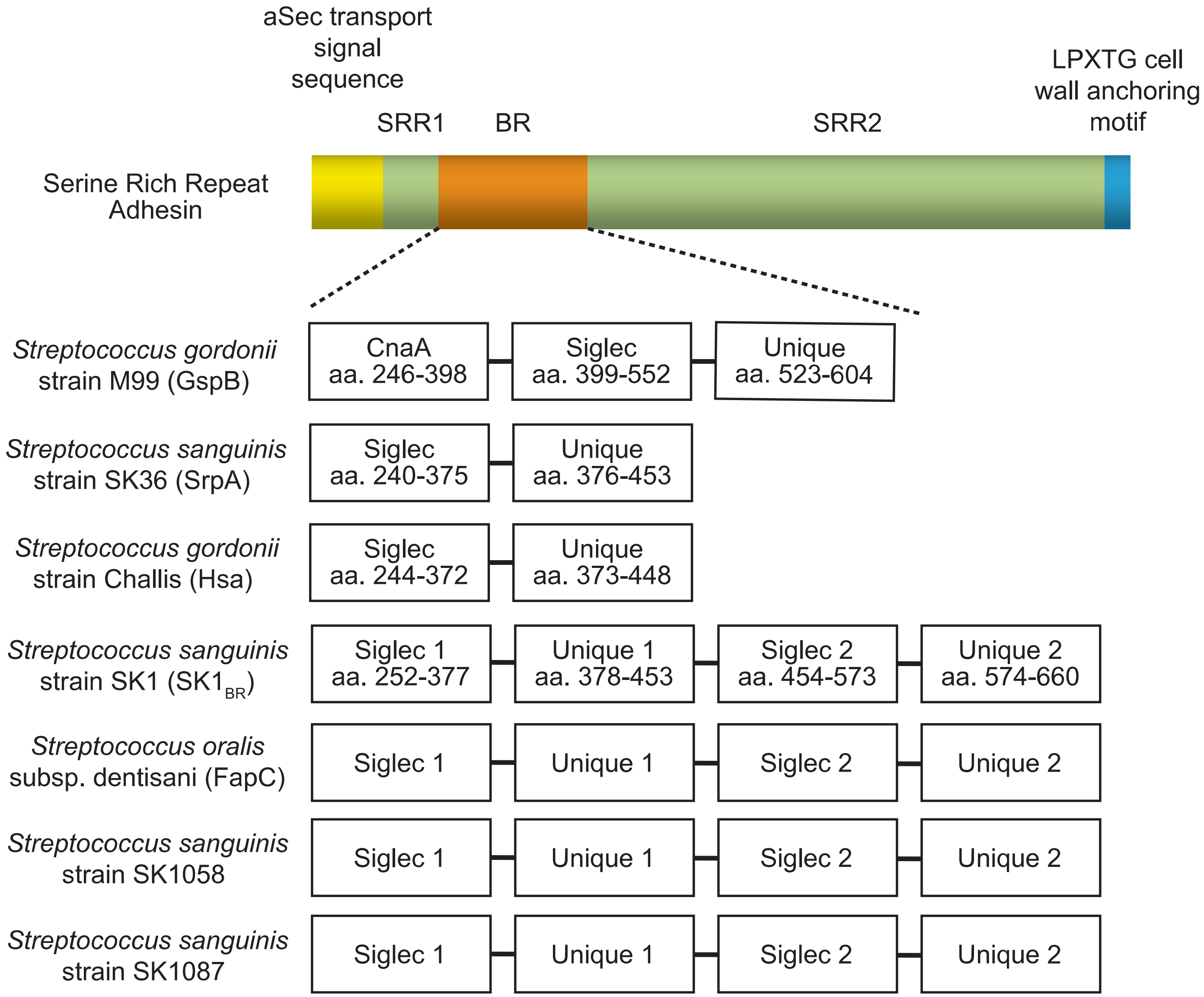

Supplementary Figure 1 Siglec-like serine rich repeat adhesins of Streptococci. Serine rich repeat proteins follow a conserved functional organization including an N-terminal signal sequence, a short serine rich repeat sequence, a binding region, a second longer serine rich repeat region, and a cell wall anchoring motif. The binding region itself consists of multiple domains and can include domains that contain diverse folds. Depicted here are the organizations of binding regions of Siglec-like SRR adhesins discussed in the text. The bacteria and strain for each binding region are listed, and if the binding region or adhesin has been given a specific name it is listed in parentheses. The SRR adhesins with tandem Siglec-like binding domains are from *S. sanguinis* strains SK160 (WP_080555651.1; 99% identity, 99% similarity), SK1058 (WP_004191732.1; 99% identity, 99% similarity), NCTC 10904 (WP_126436113; 99% identity, 99% similarity), SK1087 (WP_080558715.1; 96% identity, 97% similarity), and BCC39 D8870 (WP_125332456.1; 96% identity, 98% similarity), *S. cristatus* strain 550_SOLI (WP_141640267.1; 94% identity, 96% similarity)(6) and FapC from *S. oralis* subsp. dentisani strain F0392 (25% identity, 39% similarity) (4). Residue numbers for the domains are listed for those binding regions with reported structures (16,18,19).

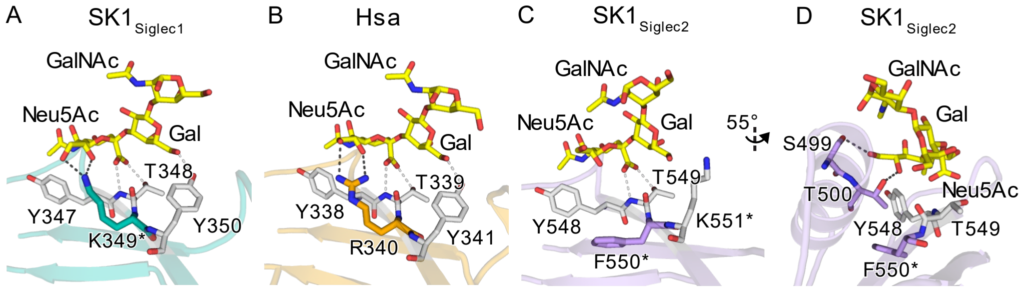

Supplementary Figure 2 Hydrogen bond contacts between the non-canonical binding motif and sTa. Transparent cartoon representations of $SK1_{Siglec1}$, Hsa (18), and $SK1_{Siglec2}$ are shown in teal, orange, and lavender respectively. sTa is shown in yellow sticks. The residues in the third position of the YTRY motif is colored A) teal, B) orange, and C-D) lavender. The other residues of the YTRY motif are shown in grey sticks. The hydrogen bonds between the third residue of the YTRY motif and sTa are shown in dark grey dashed lines and all other hydrogen bonds between sTa and the YTRY motifs are shown in grey dashed lines. (D) Residues in the CD loop of $SK1_{Siglec2}$ that hydrogen bond with sTa are shown in lavender sticks. Residues that deviate from the canonical YTRY motif definition are noted with an asterisk.

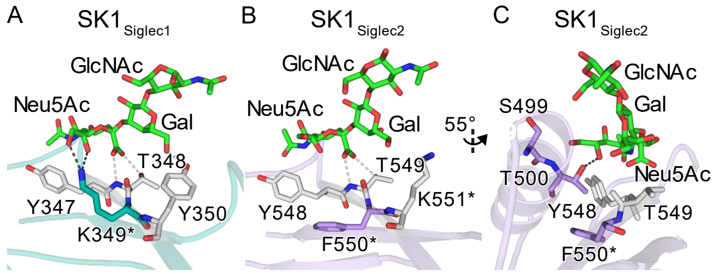

Supplementary Figure 3 Hydrogen Bond Contacts between non-canonical binding motifs of SK1$_{Siglec1}$ and SK1$_{Siglec2}$ and 3'sLn. 3'sLn is shown in green sticks bound to SK1$_{Siglec1}$ (A) and SK1$_{Siglec2}$ (B). SK1$_{Siglec1}$ and SK1$_{Siglec2}$ areshown in transparent teal and lavender cartoon respectively. The YTRY motifs of both are shown in grey sticks, except for the residue aligned with the canonical arginine, which is shown in teal and lavender for SK1$_{Siglec1}$ and SK1$_{Siglec2}$ respectively. (A) and (B) Hydrogen bond contacts between the residue aligned with the arginine are shown in dark grey dashed lines. All other hydrogen bonds between the YTRY motif and 3'sLn are shown in grey dashed line. (C) Residues in the CD loop of SK1$_{Siglec2}$ that hydrogen bond with 3'sLn are shown in lavender sticks. Hydrogen bond contacts between the CD loop and 3'sLn are shown in dark grey dashed lines. Residues that deviate from the canonical YTRY motif definition are noted with an asterisk.

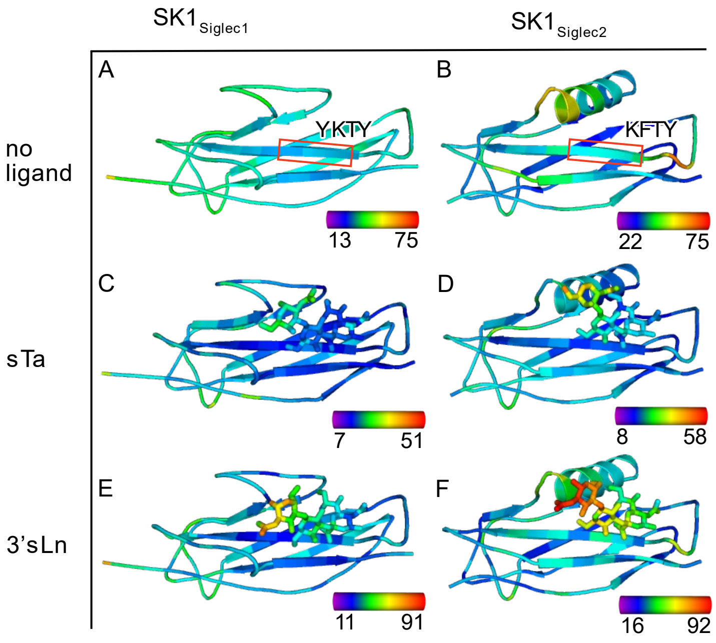

Supplementary Figure 4 Siglec domain and ligand temperature factor analysis. Here, both the ligand and the Siglec domains are colored by temperature factor. Note that the spectrum bars in the bottom right corner of each panel indicate the range of the B factors in $Å^2$.

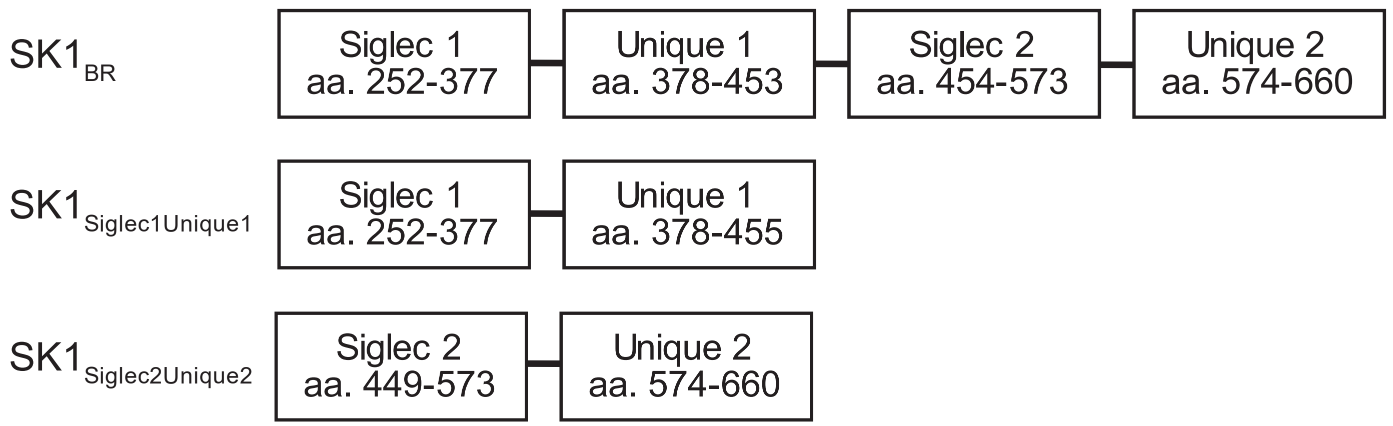

Supplementary Figure 5 Graphical representation of SK1$_{BR}$ split domain proteins. The residues of the full length adhesin included in the expressed protein are shown. Information regarding the affinity tags (not shown) is included in the Experimental Procedures section.
